# Microbiome landscape of lesions and adjacent normal mucosal areas in oral lichen planus patient

**DOI:** 10.3389/fmicb.2022.992065

**Published:** 2022-10-20

**Authors:** Jian Chen, Kaikai Liu, Xiaona Sun, Xuanxuan Shi, Guanghui Zhao, Zhongjun Yang

**Affiliations:** ^1^Department of Stomatology, Qilu Hospital (Qingdao), Cheeloo College of Medicine, Shandong University, Qingdao, China; ^2^School and Hospital of Stomatology, Cheeloo College of Medicine, Shandong University, Jinan, China; ^3^Medical Laboratory Center, Qilu Hospital (Qingdao), Cheeloo College of Medicine, Shandong University, Qingdao, China

**Keywords:** oral lichen planus, microbiome, 16S rRNA, enrichment analysis, flora imbalance

## Abstract

The pathogenesis of oral lichen planus (OLP) remains unclear, and microbial dysbiosis has been proposed to play a role in the pathogenesis of OLP. Oral mucosal swabs from 77 OLP patients and 76 healthy subjects were collected. The bacterial community among the OLP lesion, the adjacent normal mucosal, and the oral mucosal surface in healthy people were analyzed by 16S sequencing. The factor of gender and age that may affect the flora distribution of OLP patients were explored. Results indicate no significant difference in microbiota between OLP and the adjacent group. Compared with the healthy group, Neisseria, Haemophilus, Fusobacterium, Porphyromonas, Rothia, Actinomyces, and Capnocytophaga significantly increased in the OLP group. Actinomyces increased in male OLP patients, and the other six bacteria increased in female OLP patients. In female OLP patients, Lautropia and Dialister were positively correlated with age. While in male OLP patients, Moraxella, Porphyromonas, and Fusobacterium were positively correlated with age. Functional enrichment analysis suggested that abnormal energy metabolism related to ATP synthases, abnormal transport and metabolism of glycans, amino acids, and vitamins, and disorders of the local immune microenvironment might exist in OLP lesion.

## Introduction

Oral lichen planus (OLP) is a common chronic inflammatory disease of oral mucosa with an incidence of about 0.5–2%, and females between 30 and 60 years old are more vulnerable to OLP (Zhong et al., 2020). The most common types of OLP are reticulate and erosive forms, often associated with pain and discomfort in eating, swallowing, and speaking (Jung and Jang, 2022).

OLP is a chronic inflammatory disease mediated by T cells, with unclear etiology and pathogenesis. Histologically, OLP is characterized by subepithelial lymphocyte infiltration, intraepithelial lymphocytosis, and basal keratinocytes degeneration. It has been hypothesized that unknown antigens can stimulate the keratinocytes to secrete cytokines, guide the migration of T cells to the epithelium andactivate T cells, ultimately leading to the apoptosis of keratinocytes and the destruction of the basement membrane (Roopashree et al., 2010). The microbiome plays an essential role in the host immune system and helps to maintain immune homeostasis against infection at equilibrium. Changes in the species and abundance of microbiota, known as dysbiosis, is related to immune-related diseases such as rheumatoid arthritis (Alpizar-Rodriguez et al., 2020), diabetes (Al-Jameel, 2021), asthma (Tang et al., 2021), Sjogren’s syndrome (Van Der Meulen et al., 2022), and systemic lupus erythematosus (Peene and Elewaut, 2019). In recent years, dysbiosis has also been found to be associated with depression and other mental diseases (Du et al., 2020b). Mental stress is one of the predisposing factors of OLP, and dysbiosis may be related to the occurrence and development of OLP.

With the development of high-throughput sequencing technology, there have been some preliminary studies on OLP-related microorganisms. Although all studies found significant differences of the microbiota between OLP patients and healthy controls, the results varied widely (Choi et al., 2016; Wang et al., 2016, 2020; He et al., 2017; Baek et al., 2020; Deng et al., 2020; Yu et al., 2020; Du et al., 2020a). This indicates that the current study results are confusing, and more effort needs to be made for further investigation. Due to the sample size limitation, some of previous studies missed some factors that may affect microorganisms, such as age and gender of subjects. In the present study, we investigated the flora difference of microorganisms between the mucosal lesion surface and the adjacent normal mucosal surface in OLP patients and the oral mucosal surface in healthy people to find the microorganisms that might be related to OLP, and also the possible impact of age and gender factors on oral mucosa microorganisms.

## Materials and methods

### Study subjects

All subjects were recruited from the Department of Stomatology of Qilu Hospital of Shandong University (Qingdao) from January 2019 to December 2020. The inclusion criterion was OLP patients diagnosed based on the clinical and histopathological diagnostic criteria guided by the WHO (Van Der Meij and Van Der Waal, 2003). The classical clinical characteristics of OLP: reticular, erosive, atrophic, plaque-like, papular, and bullous. The classical histopathological characteristics: (1) liquefactive degeneration of the basal layer, (2) band-like dense inflammatory infiltrate of T lymphocytes, (3) normal epithelial maturation, saw-toothed anatomical prominences, Civatte bodies, and hyperkeratosis. The most common characteristic of OLP lesions in the buccal mucosa is reticular. Buccal mucosal lesions were selected for the study. The erosive OLP showed erosion or ulceration on the oral mucosa, and the non-erosive OLP was selected according to the typical clinical presentation of papules, plaques, and radiating grayish-white Wickham striae, separately or in combination (Sun et al., 2021).

The exclusion criteria were as follows: (a) receiving any treatment for OLP in the previous 3 months; (b) using prescription drugs including antibiotics or glucocorticoids for last 3 months; (c) other known oral mucosal disease; (d) severe systemic diseases; (e) pregnancy or lactation; (f) diagnosed as periodontal disease, untreated caries and more than three missing teeth. Seventy-seven OLP patients were selected for the study, including 38 females and 39 males, with a mean age of 50.97 ± 10.97 years old. The healthy subjects were selected randomly and matching the age and gender distribution of the corresponding OLP patients. Exclusion criteria were the same as those for OLP patients. Seventy-six oral health volunteers were selected for the study, including 42 females and 34 males. The mean age was 48.43 ± 4.72 years old, as shown in Table 1, which indicated that no significant difference between the two groups (*p* = 0.065). There was no significant difference in sex distribution between the two groups (*p* = 0.464) either. The group’s age and sex interference were excluded, and the selected subjects were suitable for further comparative analysis.

**Table 1:** Clinical data of OLP patients and oral health control population.

	OLP (*n* = 77)	Healthy (*n* = 76)	Value of *p*
*Age (years, mean ± SD)*	50.97 ± 10.97	48.43 ± 4.72	0.065
*Gender (n, %)*			0.464
Female	38 (49.4)	42 (55.3)	
Male	39 (50.6)	34 (44.7)	
*Erosive (n, %)*			
With	35 (45.5)		
Without	42 (54.5)		

The demographic and clinical data of all subjects were shown in Supplementary Table S1. The ethics committee of Qilu Hospital of Shandong University (Qingdao) approved the study. The approval number of the ethics is KYLL-ks-qdql201s8023. All the subjects signed informed consent prior to be enrolled in this study.

### Sample collection

After gently rinsing with water, samples were obtained by rotating a cotton swab pressed to the buccal diseased mucosa and adjacent seemingly normal buccal mucosa of patients with OLP and the buccal mucosa of healthy controls. We defined the location of 2 cm outside of the OLP lesion as the healthy adjacent tissue. Swabs were stored in test tubes and frozen at −80°C for further analysis. Swap samples were divided into three groups: OLP, adjacent, and healthy groups, respectively, based on the experimental design.

### DNA extraction, PCR amplification, and library construction

Genomic DNA of the samples were extracted using a DNA extraction Kit (Magen, Cat. No. D6356-02). Region 16S V3-V4 was selected for PCR amplification. The primers were as follows: 343F: 5′- TACGGRAGGCAGCAG -3′, 798R: 5′- AGGGTATCTAATCCT-3′. Using 50 ng of genomic DNA as a template, PCR amplification was performed through Tks Gflex DNA Polymerase (Takara, Cat. No. R060B). The PCR products were detected by electrophoresis and purified using magnetic beads. We added 20 μl of fully mixed AMPure XP beads (Beckman) to the U-type plate, and then PCR products. The mixtures were placed 5 min on the magnetic frameand wash the products with 200 μl freshly prepared 80% ethanol after discarding supernatant. And then these above steps were repeated once. We added 25 μl H_2_O for 5 min after drying at room temperature, placed 5 min on a magnetic frame, and transferred the 20 μl supernatant into a new PCR tube. The purified PCR products were used as templates for the second round of PCR amplification as the described above, and then magnetic beads were used purified the products again. PCR products were quantified by using Qubit dsDNA Assay Kit (Life Technologies, Cat. No. Q32854).

### 16S rRNA gene sequencing and data preprocessing

Raw data was obtained after sequencing the PCR products using novaseq 6,000 (Illumina, United States). Sequences were scanned by sliding window to remove raw data sequences with low quality using Trimmomatic (version 0.35) software. The raw data was spliced through Flash (version 1.2.11; Reyon et al., 2012) to get completed paired-end sequences. Sequences containing N bases, single-base repeats greater than eight, and lengths less than 200 bp in the paired-end sequence were removed using split_libraries (version 1.8.0) to get clean tags sequences. Chimeras from the clean tags were removed using the UCHIME (version 2.4.2; Edgar et al., 2011), and the valid tags sequences were obtained for subsequent analysis.

### Data analysis

Vsearch (version 2.4.2) was used to classify OTU for valid tags according to 97% similarity, and the sequence with the largest abundance in each OTU was selected as the representative sequence. RDP classifier Naive Bayesian classification algorithm was used to compare the representative sequence with the Silva (V132) database to obtain the annotation information of OTU. According to the number of sequences of each OTU in each sample, the abundance matrix of OTU in each sample was constructed. All samples were randomly selected according to the minimum depth to obtain the OTU table. After OTU classification, OTU category, OTU annotation information, and representative sequences were statistically analyzed to obtain the relative quantitative abundance of microorganisms in all samples.

### Statistical analysis

The Mann–Whitney U test was performed to compare the age impact between OLP patients and healthy control. Pearson’s chi-squared test was used to analyze the gender distribution among groups. In the subgroup analysis, the difference analysis between the two groups was Wilcoxon or student’s t-test, and the difference among the three groups was Kruskal Wallis or one-way ANOVA analysis. Other statistical methods used here were elaborated in the results section.

## Results

### Tags and OTU distribution

**Figure 1:**
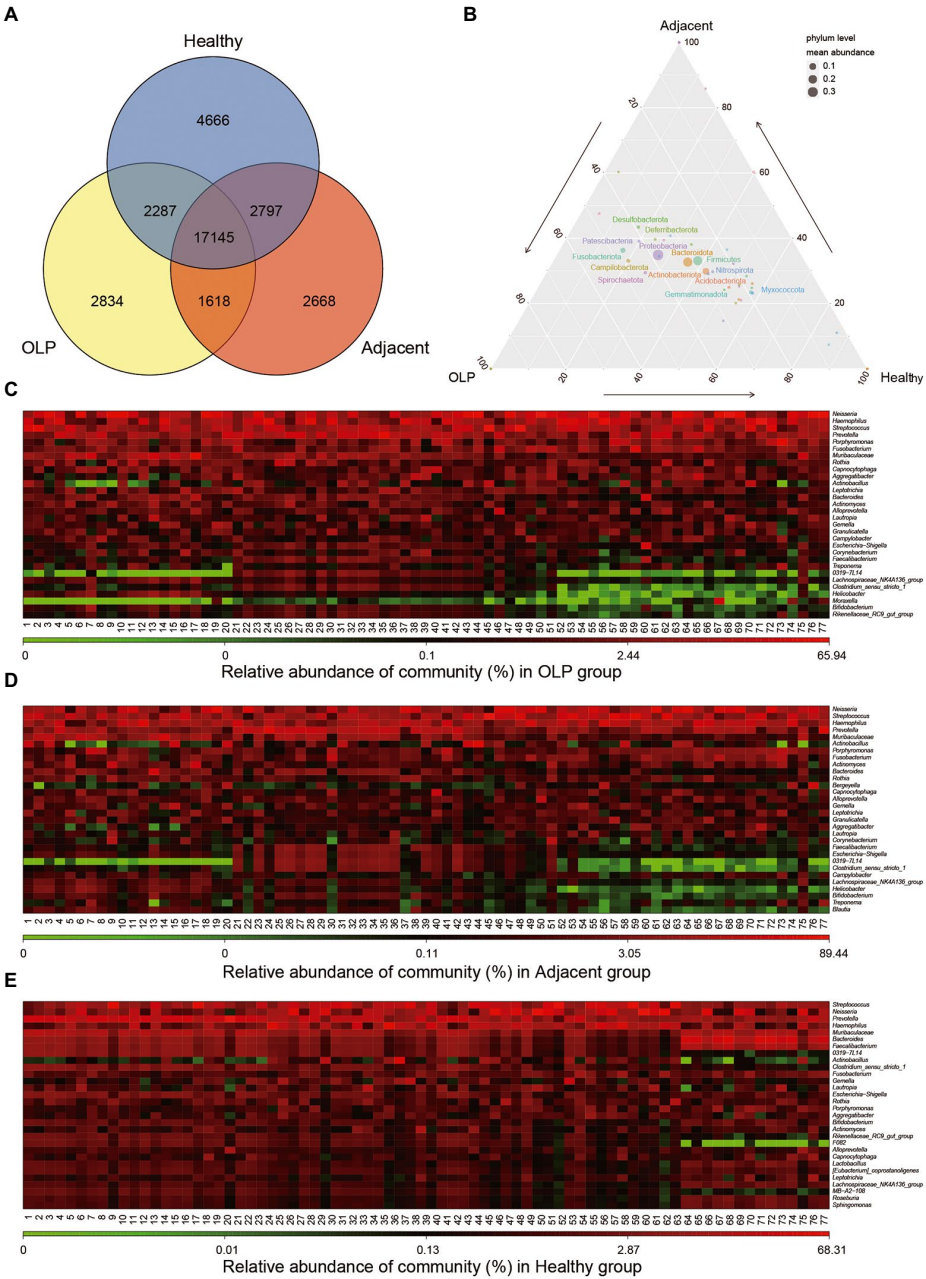
Distribution of OTU and microbiological Community. **(A)** Total number of annotated OTUs in three groups. OLP stands for oral lichen planus patients, Adjacent represents the normal mucosal area adjacent to the lesion area in OLP patients, Healthy stands for people with oral health. **(B)** Ternary Plot. The three corners represent three groups of samples, and the circle size represents the average relative abundance of the species. **(C)** Heatmap (without sample clustering) of the abundance of the TOP30 species at Genus level in the OLP group. **(D)** Heatmap (without sample clustering) of the abundance of the TOP30 species Genus level in the Adjacent group. **(E)** Heatmap (without sample clustering) of the abundance of the TOP30 species Genus level in the Healthy group.

Two hundred thirty samples were collected in total in the present study. The clean tags ranged between 26,603 and 75,454 bp after quality control. The valid tags resulting from chimera removal of clean tags ranged from 21,421 to 69,869 bp. The average length of valid tags ranged from 396.08 to 424.9 bp. The number of OTU in each sample was distributed between 606 and 5,469 bp. The details were shown in Supplementary Table S2. Venn plots of OTU were constructed in groups shown in Figure 1A. The three groups shared 17,145 OTU, which suggested that these OTUs might be conserved across all populations.

### Sample community structure distribution

Microbial abundance was obtained by annotating all samples at the level of phylum, class, order, family, genus, and species (Supplementary Table S3). Ternary Plot was drawn at phylum level using ggtern package of the R language, as shown in Figure 1B. The plot presented the difference between the dominant species among the three samples and showed the proportion of different species. At the genus level, bacteria with the TOP 30 abundance were selected to draw a heat map, as shown in Figures 1C–E. The results showed that the bacteria enrichment pattern in healthy controls was consistent and significantly different from that in OLP patients. Bacteria in the OLP group and adjacent group demonstrated the same abundance change. Further sample clustering analysis at the phylum and family level showed that heterogeneity within the OLP group became more complex (Supplementary Figures S1A,B).

### Analysis of bacterial diversity

#### Alpha diversity

**Figure 2:**
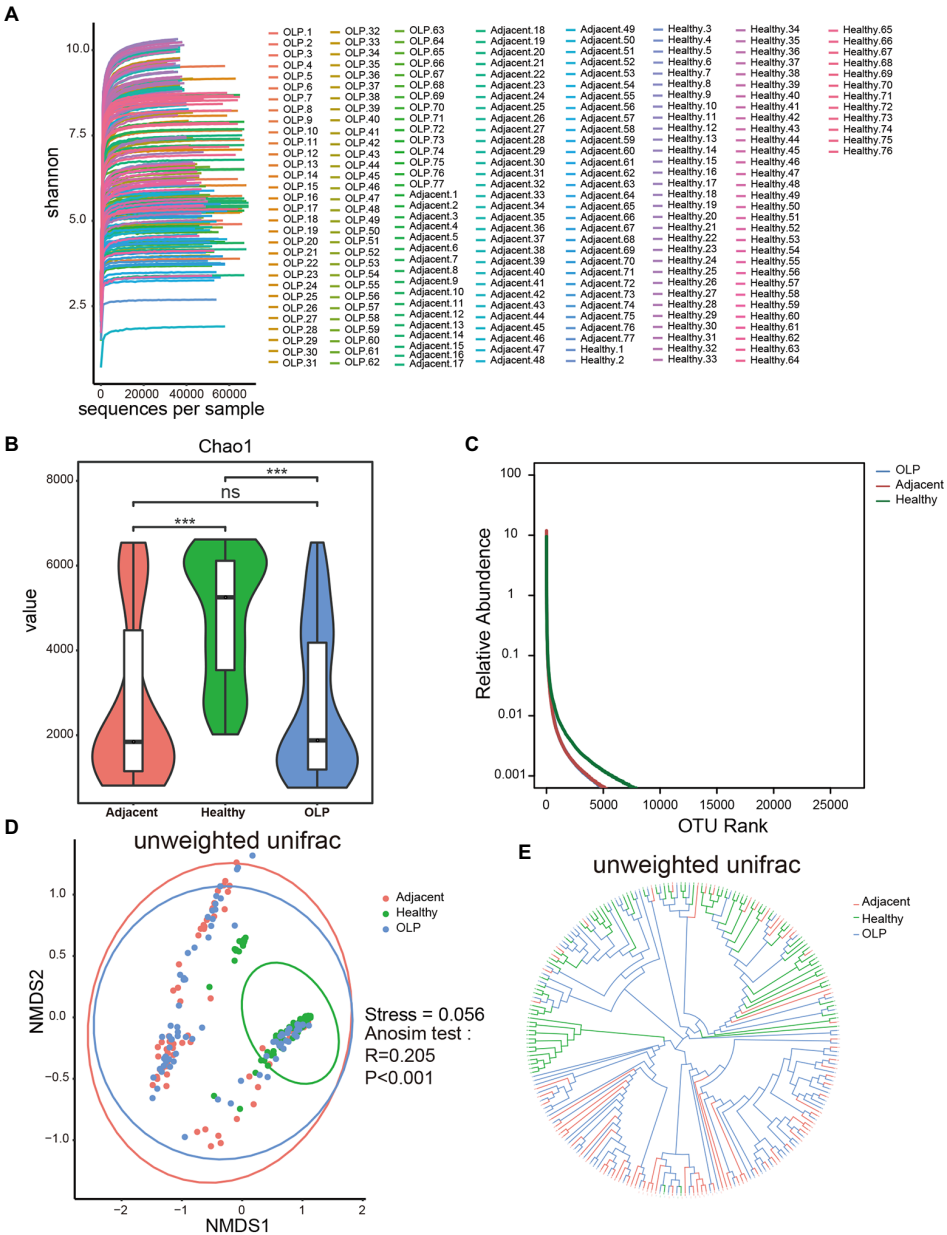
Alpha diversity and beta diversity of species in the three groups. **(A)** Dilution curve of Shannon index. **(B)** The violinplot of Chao1 index. Groups are colored on the horizontal axis, and index values are indicated on the vertical axis. The asterisks represented the statistical *p* value, *p < 0.05, **p < 0.01, ***p < 0.001. **(C)** Rank Abundance curve. The horizontal axis represents the ranking of OTU and the vertical axis represents the relative abundance of OTU. Species richness is reflected by length along the horizontal axis, and species evenness by the shape of the curve. **(D)** NMDS analysis. The horizontal axis (NMDS1) and the vertical axis (NMDS2) are the sorting axes, and each point represents a sample. The statistical method was anosim test based on unweighted unifrac distance. **(E)** Sample hierarchical clustering analysis based on unweighted Unifrac distance.

Dilution curves shown in Figure 2A tended to be horizontal for all samples, which indicated that the sequencing depth was sufficient and the downstream analysis results were reliable. Five indexes including chao1, shannon, simpson, PD whole tree, and observed species index were calculated to evaluate the alpha diversity as shown in Supplementary Table S4; Figure 2B; and Supplementary Figure S2. The results of the five indexes all showed that compared to the healthy group, species richness and diversity were significantly decreased in the OLP group and adjacent group, while there was no significant difference between the OLP group and adjacent group. Rank Abundance was another calculation method that could simultaneously explain the abundance and evenness of species in the samples. As shown in Figure 2C, the horizontal axis span of the healthy group was more significant, also indicating that the oral species diversity of the healthy group was more extensive than that of the OLP group.

#### Beta diversity

The comparison of beta diversity from each group was plotted by non-metric multidimensional scaling (NMDS; Figure 2D). With each dot representing one sample, the healthy group was essentially able to cluster, although a few samples showed outlier characteristics. The OLP patients showed distinct discrete characteristics, suggesting that there may be widespread heterogeneity among the OLP patients. The Stress coefficient was 0.056, which meant an excellent sorting (Stress <0.1).

UPGMA was another analysis method that reflected beta diversity. The closer the branches were, the more similar the samples were. The results, shown in Figure 2E, confirmed the above results by showing that the healthy group could be clustered together.

### Analysis of different species among different groups

#### Overall difference analysis

Non-parametric multivariate analysis of variance Adonis and non-parametric similarity analysis Anosim analyzed the overall difference between the OLP, adjacent, and the healthy groups. Five distance algorithms, including Binary Jaccard, Bray Curtis, Euclidean, Unweighted unifrac, and Weighted unifrac, all showed significant differences among the three groups (*p* < 0.01). The R^2^ value of Adonis analysis or the R-value of Anosim analysis explained the difference between samples. The similarities between the OLP and the adjacent groups may affect the results. As shown in Table 2, when the OLP and the healthy groups were analyzed separately, the R^2^ and R values increased to a certain extent.

**Table 2:** Adonis test and Anosim test between multiple groups.

	OLP vs. adjacent vs. Healthy	OLP vs. Healthy	OLP female vs. OLP male	With erosive vs. without erosive
*Adonis test*				
Binary jaccard	*R*^2^ = 0.048, *p* < 0.001	*R*^2^ = 0.052, *p* < 0.001	*R*^2^ = 0.018, *p* = 0.058	*R*^2^ = 0.010, *p* = 0.941
Bray curtis	*R*^2^ = 0.065, *p* < 0.001	*R*^2^ = 0.074, *p* < 0.001	*R*^2^ = 0.013, *p* = 0.358	*R*^2^ = 0.012, *p* = 0.460
Euclidean	*R*^2^ = 0.029, *p* < 0.001	*R*^2^ = 0.027, *p* < 0.001	*R*^2^ = 0.012, *p* = 0.457	*R*^2^ = 0.011, *p* = 0.527
Unweighted unifrac	*R*^2^ = 0.072, *p* < 0.001	*R*^2^ = 0.081, *p* < 0.001	*R*^2^ = 0.020, *p* = 0.079	*R*^2^ = 0.009, *p* = 0.896
Weighted unifrac	*R*^2^ = 0.059, *p* < 0.001	*R*^2^ = 0.075, *p* < 0.001	*R*^2^ = 0.008, *p* = 0.651	*R*^2^ = 0.016, *p* = 0.247
*Anosim test*				
Binary jaccard	*R* = 0.197, *p* < 0.001	*R* = 0.295, *p* < 0.001	*R* = 0.030, *p* = 0.073	*R* < 0.001, *p* = 0.951
Bray curtis	*R* = 0.139, *p* < 0.001	*R* = 0.216, *p* < 0.001	*R* = 0.001, *p* = 0.405	*R* < 0.001, *p* = 0.918
Euclidean	*R* = 0.041, *p* < 0.001	*R* = 0.052, *p* < 0.001	*R* < 0.001, *p* = 0.449	*R* < 0.001, *p* = 0.752
Unweighted unifrac	*R* = 0.205, *p* < 0.001	*R* = 0.297, *p* < 0.001	*R* = 0.025, *p* = 0.097	*R* < 0.001, *p* = 0.954
Weighted unifrac	*R* = 0.082, *p* < 0.001	*R* = 0.142, *p* < 0.001	*R* < 0.001, *p* = 0.750	*R* < 0.001, *p* = 0.669

#### Difference analysis between the subgroups

Subgroup analysis of OLP patients according to gender was further conducted, as shown in Table 2, suggesting no difference in the diversity of enriched flora between males and females in OLP patients. The presence or absence of erosion was often used for the clinical classification of OLP patients. Further subgroup analysis of OLP patients according to the erosive and non-erosive groups also showed no difference in the diversity of enriched flora.

#### Screening of different species among the three groups

**Figure 3:**
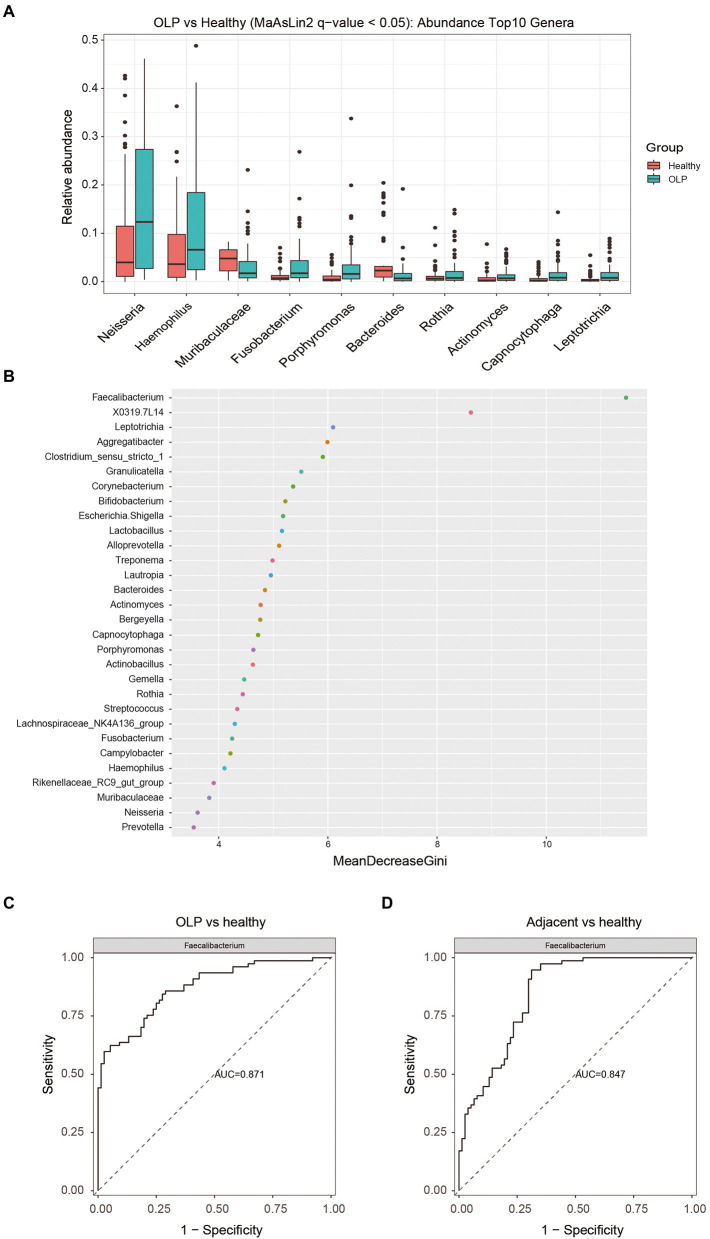
Screening of differential species and analysis of their importance. **(A)** Top 10 different species at genus level among the three groups. The colors represent different groups, and the vertical axis represents log transitions of relative abundance of species. **(B)** Random forest analysis. The horizontal axis is the measure of importance, and the vertical axis shows the species in order of importance. **(C)** The ROC plots of Faecalibacterium (OLP vs healthy). **(D)** The ROC plots of Faecalibacterium (Adjacent vs healthy).

Bacteria with significant differences between OLP and healthy groups at phylum, class, order, family, and genus level were shown in Supplementary Table S5 by MaAslin2 method. At the genus level, bacteria with TOP10 abundance and *q* < 0.05 were selected to draw a boxplot, as shown in Figure 3A. *Neisseria*, *Haemophilus*, *Fusobacterium*, and *Porphyromonas* were significantly enriched in the OLP group. *Bacteroides* and *Muribaculaceae* specifically increased in the healthy group. The Wilcoxon method was applied to the differential analysis of the OLP vs. Adjacent comparison group. The data from CLR normalized relative abundance were also used for analysis. The different species were screened by *q* value <0.25 (*q* < 0.05 could not screen for the difference), and the two genera that met the conditions were displayed by boxplots. The results were shown in Supplementary Figures S3A,B.

To eliminate the interference caused by the comparison between the three groups, we re-compared the OLP group and healthy group, as shown in Supplementary Figures S3C,D. As expected, significant alpha diversity and beta diversity existed between the two groups.

#### Screening of different species between genders

Although there was no significant difference in the diversity of flora between genders in the OLP group, gender was still an essential factor between different groups. Comparing each gender between different groups could eliminate some possible interfering factors, such as hormone levels. The three groups of males and females were compared then in our study respectively, as shown in Supplementary Figures S4, S5. The results showed that compared to the healthy female group, the bacterial diversity of OLP female patients was also significantly reduced. *Neisseria*, *Haemophilus*, *Porphyromonas*, *Fusobacterium*, *Rothia*, and *Capnocytophaga* increased, while *Prevotella*, *Muribaculaceae*, and *Bacteroides* decreased in OLP female patients.

Interestingly, among the different bacteria with the TOP10 abundance, nine genera of OLP male patients showed a significant decrease compared to the healthy male group, including *Bacteroides*, *Faecalibacterium*, *Lactobacillus*, *Clostridia_UCG-014*, *Sphingomonas*, *Roseburia*, *coprostanoligenes* and *Lachnospira*. However, *Actinomyces* increased in OLP male patients. These results further suggested the necessity of evaluating the gender impact among the three groups. In the future study, such gender impact needs to be verified in a larger population, and the underlying mechanism responsible for such differences should be explored.

#### Correlation between different species and age in OLP group

The correlation between age and bacteria in the OLP group, OLP female patients, and OLP male patients at the genus level was analyzed to investigate whether age was another factor affecting the bacterial diversity in OLP patient. The results were presented in Supplementary Table S6. *Moraxella* and *Campylobacter* were positively correlated with age in all OLP patients, while *Haemophilus* was negatively correlated with age. In OLP female patients, *Lautropia* and *Dialiste* were positively correlated with age, while *Haemophilus* was negatively correlated with age. In OLP male patients, *Moraxella*, *Porphyromonas*, and *Fusobacterium* were positively correlated with age, while *Helicobacter*, *Muribaculaceae*, *Clostridium sensu stricto 1*, *Lachnospiraceae NK4A136 group*, and *Alistipes* were negatively correlated with age.

#### Random forest analysis

Random forest was a machine learning method, first proposed by Leo Breiman and Adele Cutler, which could effectively and accurately classify microbial community samples and find out the key species that could distinguish the differences between different groups. The results of ROC curve analysis was shown in Supplementary Table S7. The genera with the TOP30 relative abundance in the three groups were selected to draw a dot map using the randomForest package of the R language, as shown in Figure 3B. The value of *Faecalibacterium* was most remarkable. The ROC plots of *Faecalibacterium* were shown in Figures 3C,D. The TOP 10, TOP 20, and TOP 30 taxa of the ROC curve were presented in Supplementary Figures S6A–F.

#### PICRUSt analysis

PICRUSt function prediction analysis predicted the functional composition of known microbial genes based on 16S sequencing data annotated in the Greengenes database.

### KEGG function prediction based on 16S

**Figure 4:**
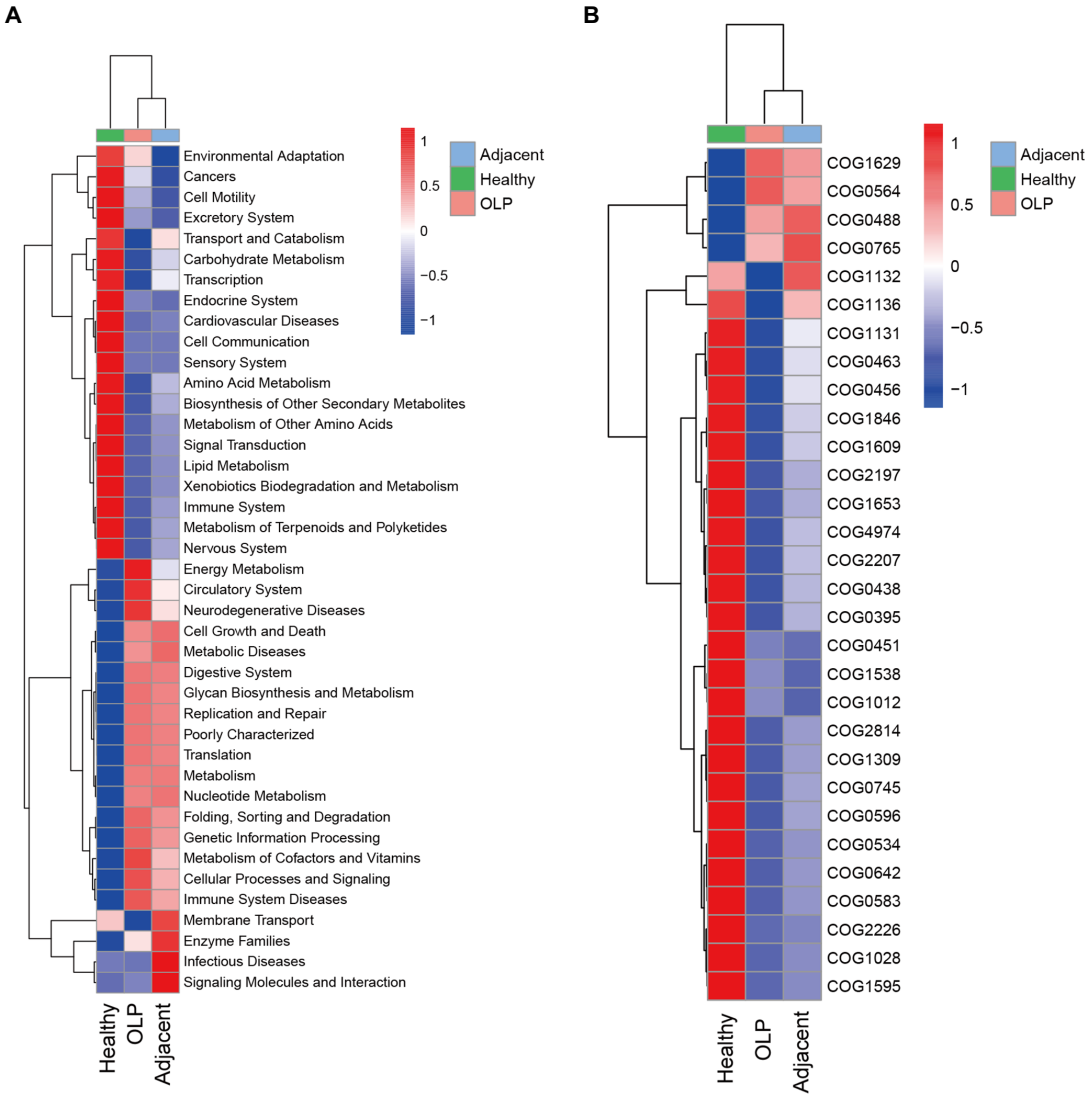
Functional prediction results. **(A)** Function prediction results t level 2 based on KEGG database. **(B)** COG distribution among the three groups.

Kyoto Encyclopedia of Genes and Genomes (KEGG) is a genomic and functional information database. According to the Kruskal-Wallis algorithm, differential statistics of the KEGG predictions were performed on level 2. The heatmap was plotted and shown in Figure 4A. The OLP group was significantly high enrichment in Energy Metabolism, Neurodegenerative Diseases, Glycan Biosynthesis and Metabolism, Metabolism of Cofactors and Vitamins, and Immune System Diseases, while low enrichment in Transport and Catabolism, Carbohydrate Metabolism, Amino Acid Metabolism, and Lipid Metabolism, which suggested that when OLP lesions appeared, the energy metabolism, the metabolism of sugars and amino acids and the local immune microenvironment might be dysregulated.

### COG function prediction based on 16S

Clusters of Orthologous Groups of proteins (COG) were lineal homologous genes’ databases. In the evolutionary process, lineal homologous genes usually had the same or similar functional characteristics. The COG orthology obtained by checking the clustered OTU representative sequence in the Greengenes database was calculated according to the Kruskal-Wallis algorithm. TOP30 of the different results were selected to draw the heatmap, as shown in Figure 4B; Supplementary Table S8. In OLP patients, pathways involving iron transport protein, pseudouridylate synthases, and ATPase components were significantly high enriched. In contrast, pathways involving glycosyltransferases relevant to cell wall biogenesis, acetyltransferases, and transcriptional regulators were low enriched. These results suggested that the microbiota enriched in OLP patients may be abnormal in iron ion transport, pseudouridine acid utilization, and cell wall integrity maintenance.

## Discussion

This study found similar microbial structures on the mucosal lesion surface and normal mucosal surface adjacent to the lesion in OLP patients. We also found different microorganisms between these OLP patients and healthy subjects people and heterogeneity of bacterial flora distribution among OLP patients. Gender and age were the factors that may affect microbiota in the OLP patients.

Oral mucosal swabs were selected to obtain the samples in in this study because the swabs are easy to obtain operate, and the oral cavity has a highly heterogeneous ecosystem. Oral bacterial communities are site-specific, and bacteria on the mucosal surface may have direct impact on mucosa more than their counterparts in saliva (Du et al., 2020a). To accurately detect the changes of oral microorganisms in OLP patients, we compared the microbiota on the mucosal lesion surface and the normal mucosal surface adjacent to the lesion in OLP patients. Similar changes occurred in the microbiota at both the lesion and adjacent sites, suggesting that the OLP disease state affects the entire oral microbiome. Meanwhile, this also may mean that the seemingly normal mucosa is still at risk of disease or even already has micro-lesions that are difficult to discern by naked eyes.

Contrary to the previous studies, our study showed α diversity of microbiota in the OLP group decreased compared to the healthy group (Choi et al., 2016; Wang et al., 2016, 2020; He et al., 2017; Deng et al., 2020; Du et al., 2020a; Yu et al., 2020). The factors contributing to the different results may include the diversity of genotypes in different regions, eating habits, types of samples, sampling methods, and sample size. According to results from previous research, the microbial diversity was slightly lower in the OLP group than in the health group, although there was no statistical difference (Deng et al., 2020). This research probably collected samples more similar to ours than others because the samples were collected in the same city. Most mucocutaneous diseases such as atopic dermatitis, psoriasis, Crohn’s disease, and ulcerative colitis exhibit decreased microbiome diversity (Alekseyenko et al., 2013; Lynde et al., 2016; Pascal et al., 2017). Changes in the local microenvironment during the occurrence of diseases lead to dysbacteriosis and the emergence of dominant flora. Some researchers believe that reducing oral bacterial diversity is associated with a high risk of oral mucosa diseases (Bescos et al., 2020). The dysbiosis of the oral microbial community, without the same specific microorganisms, was detected in OLP patients, which showed the functional aspects of oral microbiota are more critical in the development of OLP than the composition of oral microbiota (Jung and Jang, 2022).

This study showed significant differences in microbial β diversity between the OLP and the healthy groups. The flora distribution in the OLP group was widely heterogeneous compared to their healthy counterparts. The healthy group had good consistency in species enrichment except for a few samples. The cause of the sample outgroup might be due to the factors affecting the oral microenvironment, such as smoking and periodontal disease, or even that these samples were in the critical state of OLP, which required further follow-up and verification. Other studies found no significant difference in microbial β diversity between OLP patients and healthy people (Wang et al., 2016; Deng et al., 2020; Yu et al., 2020), which might be resulted from the different sample types. Previous studies in which saliva samples were collected, might be mixed with planktonic bacteria in saliva. To our knowledge, our study has the largest sample size so far about OLP-associated microbes, which could be more persuasive to claim the heterogeneity of flora distribution in OLP patients. Interestingly, another study found that while there was no difference in salivary microbial β diversity between OLP patients and healthy people, there was a significant difference in tissue microbial β diversity between these two groups (Wang et al., 2020). The microbes in saliva and tissues of OLP patients were similar but still different. Samples from different lesion depths might affect the bacteria variability. The bacterial communities in OLP tissues were characterized by decreased α diversity but increased β diversity compared to those on the mucosal surface (Baek et al., 2020). Microbes on the mucosal surface of OLP patients may enter subepithelial tissues through the damaged epithelium, possibly causing or exacerbating disease status and reducing bacterial diversity in OLP tissues, which may mean that the closer to the lesion, the less microbial diversity. The subgroup analysis of OLP patients according to the erosive and non-erosive groups showed no difference in the diversity of enriched flora in this research. Previous studies have found significantly higher abundances of the *fungi Candida* and *Aspergillus* in patients with reticular OLP and of *Alternaria* and *Sclerotiniaceae_unidentified* in patients with erosive OLP were observed compared to the healthy groups (Li et al., 2019b).

Perhaps due to the large sample size in this study, more different microbial species of OLP were found here compared to previous studies. We found that at the genus level, *Neisseria*, *Haemophilus*, *Fusobacterium*, *Porphyromonas*, *Rothia*, *Actinomyces*, and *Capnocytophaga* increased on the surface of mucosa in the OLP group. According to the current published studies, the results about *Neisseria* and *Haemophilus* distribution are still controversial. Our results are consistent with Yu’s results (Yu et al., 2020). Some studies have found the opposite results (Wang et al., 2016; Deng et al., 2020; Du et al., 2020a). Except for these two bacteria, the same results for other bacteria have been reported in previous studies (Wang et al., 2016, 2020; He et al., 2017; Deng et al., 2020; Du et al., 2020a; Yu et al., 2020). It has also shown that the presence of *Campylobacter rectus*, *Fusobacterium nucleatum* and *Neisseria mucosa* was associated with OLP, and these species are consistent with our study (Carvalho et al., 2019). Based on the current studies on OLP microbes, the results of each study are not entirely consistent, which further validates our results that OLP microbes are highly heterogeneous. Among these bacteria, Porphyromonas gingivalis lipopolysaccharide was involved in the pathogenesis of OLP (Wang et al., 2018; Zeng et al., 2018). The mechanism of other bacteria related to OLP has not been reported. However, all bacteria except *Neisseria* may be involved in oral squamous cell carcinoma (OSCC; Irfan et al., 2020). Because OLP is potentially cancerous, there is a risk that these bacteria may promote the conversion of OLP into OSCC, even if it is not clear whether these bacteria are the cause or result of OLP. Bacteria of *Fusobacterium* and *Porphyromonas* are mainly studied so far, both of which can promote the occurrence and development of OSCC through various mechanisms (Li et al., 2020; Mcilvanna et al., 2021; Vyhnalova et al., 2021). The dominant bacteria of OSCC were different in different regions and stages (Zhang et al., 2019). Further research is warranted to evaluate whether there is a similar situation as OSCC in OLP.

This study also compared the microflora of OLP patients and healthy subjects by gender. Although there was no significant difference in the diversity of flora between males and females in OLP patients, the flora distribution was different between genders. The results showed a significant increase in *Neisseria*, *Haemophilus*, *Porphyromonas*, *Fusobacterium*, *Rothia*, and *Capnocytophaga* in female OLP patients and *Actinomyces* in male OLP patients, suggesting that gender affects OLP microorganisms. Hormones may be one of the essential factors contributing to these results. Microbial exposure and sex hormones exert potent effects on autoimmune diseases, which are more prevalent in women than men (Markle et al., 2013). Although OLP is not an autoimmune disease, it is associated with immune disorders and is more common in women, especially perimenopausal women (Mohan et al., 2017). In the absence of estrogen, the oral microbiota changes and decrease in estrogen levels in perimenopausal women are more likely to trigger depression and lead to OLP (Mohan et al., 2017). Estrogen can enhance oral epithelial cell barrier formation (Choi et al., 2018). Decreased estrogen levels can lead to oral epithelial atrophy, which is prone to OLP (Ciesielska et al., 2021). The mean serum levels of FSH and LH are significantly higher in OLP female patients. The high serum levels of FSH and LH can affect OLP pathogenesis because of estrogen and progesterone modulation (Lavaee et al., 2021). In the absence of estrogen, the dynamics of oral microbiota is changed (Lucisano et al., 2021). Hormone levels were not included in our study, which certainly needs to be verified in the future studies. However, there were no significant differences in the microbiota between healthy men and women, which possibly indicated that hormones alone might not exert significant effect on our parameters set in this study. This study found that *Moraxella* and *Campylobacter* were positively correlated with age in OLP patients, while *Haemophilus* was negatively correlated with age. It should be noted that the age of the OLP group showed a right-biased distribution, and it was difficult to fully match the age structure of the OLP group when choosing the healthy controls. Most healthy controls were distributed between 40 and 60 years old and showed an approximately normal distribution. In addition, patients of different genders had different flora with age, which further indicated that age and gender impacted OLP microbes. Aging process is associated with a low-grade systemic inflammatory status, even in the absence of clinical signs of infections, which might interfere with the resident microbial population of the oral cavity (Hajishengallis, 2014). Among the subgingival periodontal microbiota, the content and proportion of *Actinomyces* arehigher in the elderly group (Feres et al., 2016). In addition, the decline of function in the immune system brought on by biological age advancement generally referred to as ‘immunosenescence’, which also contribute to the increased susceptibility of elderly individuals to microbial infections (Feres et al., 2016). Different dietary habits of ages and genders also affect the distribution of oral microbes (Jovicic, 2015). Our study suggests that age and gender influence the distribution of OLP microbes, which reminds us that future studies on OLP microbes may require more control and comparison of variables.The result of random forest analysis showed that *Faecalibacterium* made the more outstanding contribution to the grouping. Compared to the healthy group, *Faecalibacterium* was less enriched in OLP and adjacent groups, with AUC values of 0.871 and 0.847, respectively. *Faecalibacterium* is one of the enteric commensal bacteria belonging to the Clostridium leptum group, which has systemic anti-inflammatory properties and supports mucosal immune homeostasis, plays a vital effect on oral and system healthy (Hornef and Pabst, 2016; Xu et al., 2021). More tooth loss was significantly associated with the lower relative abundance of *Faecalibacterium* independent of multiple covariates (Xu et al., 2021). The richness and quantity changes of *Faecalibacterium* have also been observed in systemic diseases such as Gastrointestinal disorders, Metabolic syndromes, Nervous system diseases and renal diseases (Leylabadlo et al., 2020).

PICRUSt functional prediction analysis was also conducted in this study. The results suggested that the OLP patients had local immune microenvironment disorder, abnormal energy metabolism, abnormal metabolism of carbohydrates, amino acids, lipids, and other nutrients. Metabolites were differentially expressed in serum and urine of OLP patients. These metabolites participated in many pathological processes, including inflammation lesion, carbohydrate metabolism disorder, and abnormal energy expenditure (Yang et al., 2017, 2020; Li et al., 2019a). Most of the differentially expressed metabolites identified in the serum sample were subgrouped as lipids and lipid-like molecules (Yang et al., 2017). Another study showed that the content of glutamic acid decreased in the OLP patients, leading to OLP (Xin et al., 2021).

Our study has the following characteristics compared to previous studies of OLP and microbiota. First, this research is the most extensive study of bacterial flora diversity in oral lichen planus according to the current PUBMED query. We collected the oral microbiome from 77 OLP and 76 healthy volunteers. Second, we found heterogeneity in the flora distribution among patients with lichen planus, and the different species were associated with sex and age. Third, functional enrichment analysis suggested that there might be abnormal energy metabolism related to ATP synthases in the diseased area, abnormal transport and metabolism of glycans, amino acids and vitamins, and disorders of the local immune microenvironment. There are also some limitations to this study. First, the inclusion criteria were broad and did not exclude or consider possible risk factors such as smoking and periodontal disease for further analysis. Second, the age of the selected population was mainly concentrated in 40 to 60 years old, which is relatively narrow. The microbial information of the younger or older population cannot be reflected in terms of age-microbial correlation. Third, this study lacks further validation processes related to OLP and microorganisms and only describes the objective fact of dysbiosis in OLP.

## Conclusion

There may be many factors affecting oral microbiome in OLP patients. The results of this study show that age and gender may have significant impact on it. The significance of studying OLP microbes remains not only in discovering the underlying causes of OLP but also in finding new ways to prevent and possibly treat it. Different bacteria in oral mucosa between OLP and non-OLP patients with the same background may be the cause or important diagnostic marker of OLP. Oral microbiome may become a target for disease prevention and treatment. However, the current research on OLP-related microbes is relatively superficial. The results from variety of studies are not consistent, and therefore more studies are needed to achieve this goal.

## Data availability statement

The data presented in this study have been deposited into Genome Sequence Archive (GSA) https://bigd.big.ac.cn/gsa/browse/CRA008410: accession number is CRA008410.

## Ethics statement

The studies involving human participants were reviewed and approved by Ethics Committee of Qilu Hospital (Qingdao) Shandong University. The patients/participants provided their written informed consent to participate in this study. Written informed consent was obtained from the individual(s) for the publication of any potentially identifiable images or data included in this article.

## Author contributions

ZY designed and conceived the study and proofread the manuscript. JC, KL, XSu, and XSh collected the specimens. JC and KL performed the experiments. GZ analyzed the data. JC and XSu drafted the manuscript. All authors contributed to the article and approved the submitted version.

## Funding

This research is supported by the Chinese National Natural Science Foundation Projects grant no. 81802068, Key projects of Qingdao Science and Technology plan grant no. 20–3-4-40-nsh.

## Conflict of interest

The authors declare that the research was conducted in the absence of any commercial or financial relationships that could be construed as a potential conflict of interest.

## Publisher’s note

All claims expressed in this article are solely those of the authors and do not necessarily represent those of their affiliated organizations, or those of the publisher, the editors and the reviewers. Any product that may be evaluated in this article, or claim that may be made by its manufacturer, is not guaranteed or endorsed by the publisher.
