# Supplementary material for: Microbiome landscape of lesions and adjacent normal mucosal areas in oral lichen planus patient
Source: Front Microbiol. 2022 Oct 20;13:992065. doi: 10.3389/fmicb.2022.992065 (PMC9630593; doi:10.3389/fmicb.2022.992065)
Supplement: Supplementary file 1 [file Image_1.PDF]

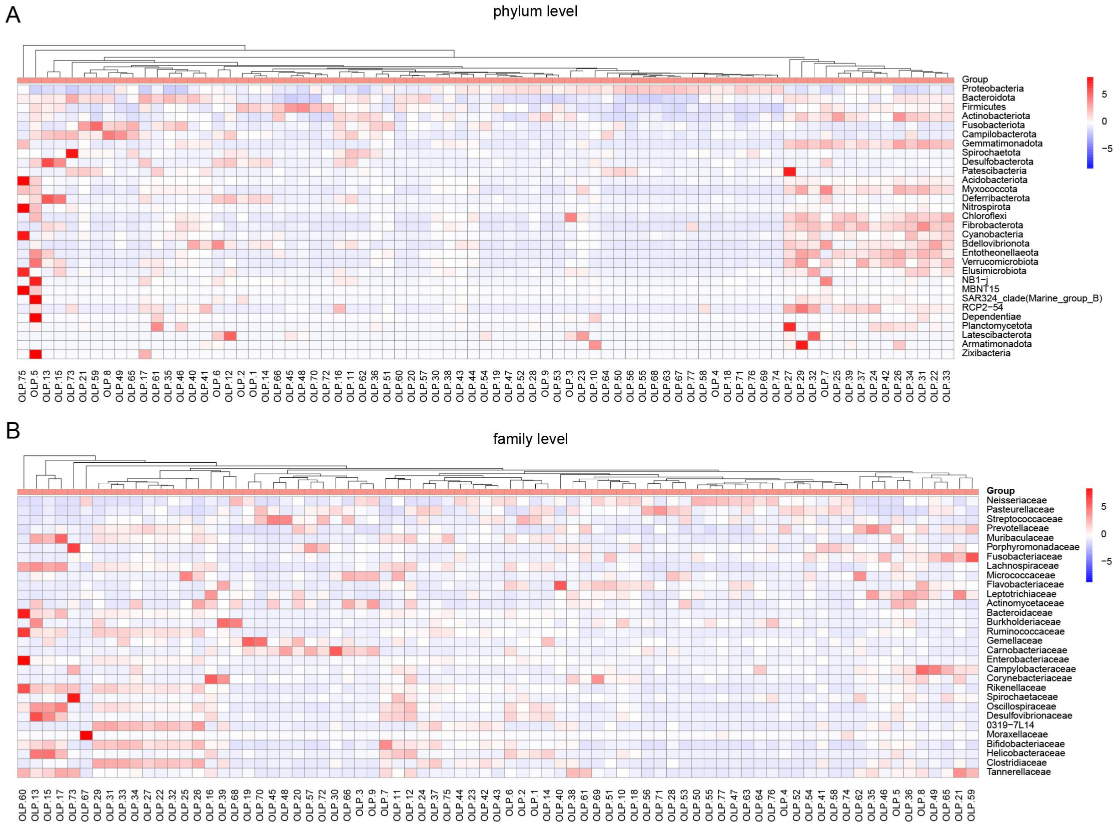

Figure S1 Clustering analysis at the Phylum and Family level within the OLP group. (A) Heatmap (with sample clustering at Phylum level) of the abundance of the TOP30 species in the OLP group. (B) Heatmap (with sample clustering at Family level) of the abundance of the TOP30 species in the OLP group.

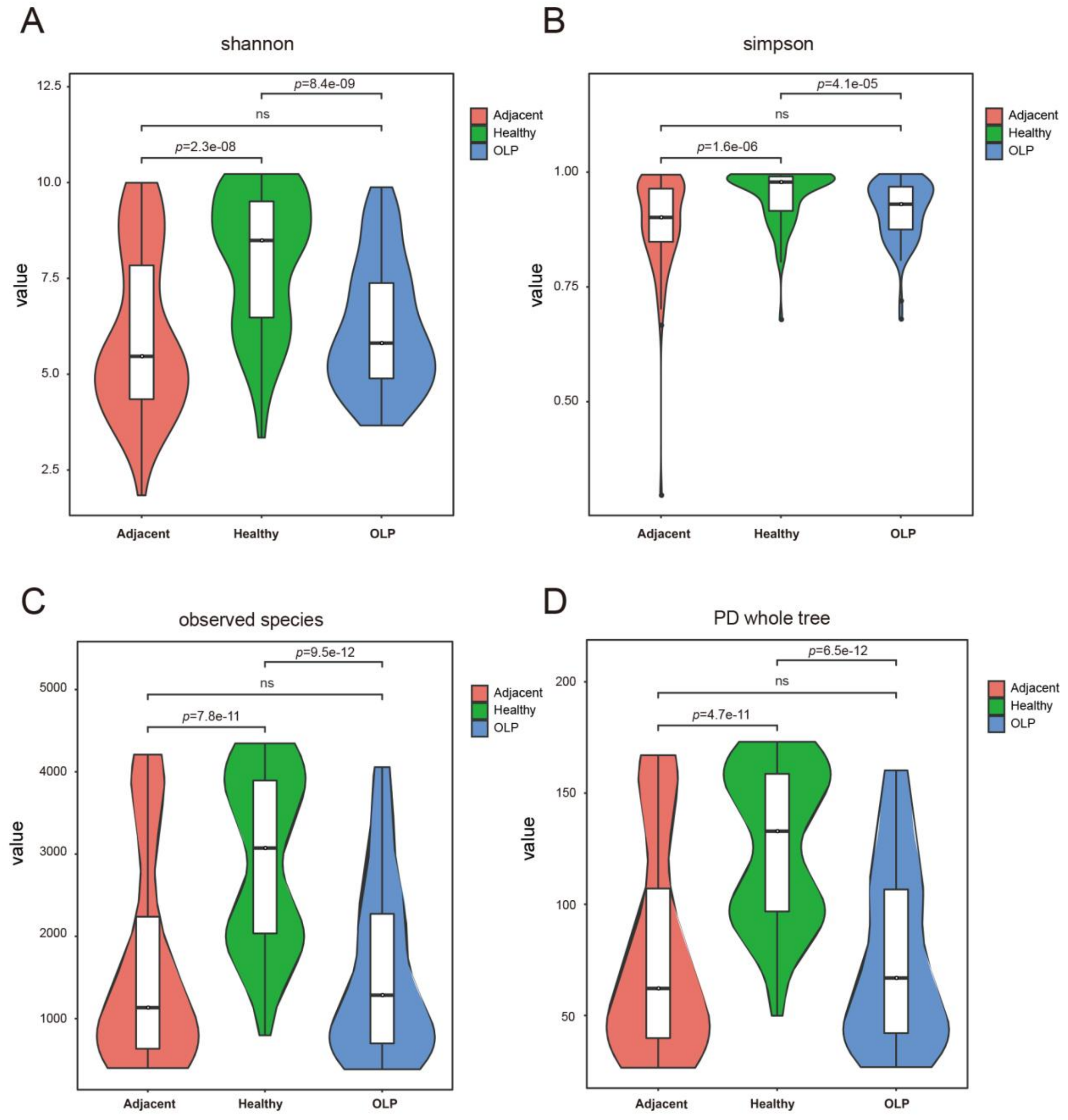

Figure S2 Alpha diversity index. (A) Shannon index. (B) Simpson index. (C) Observed species index. (D) PD whole tree index.

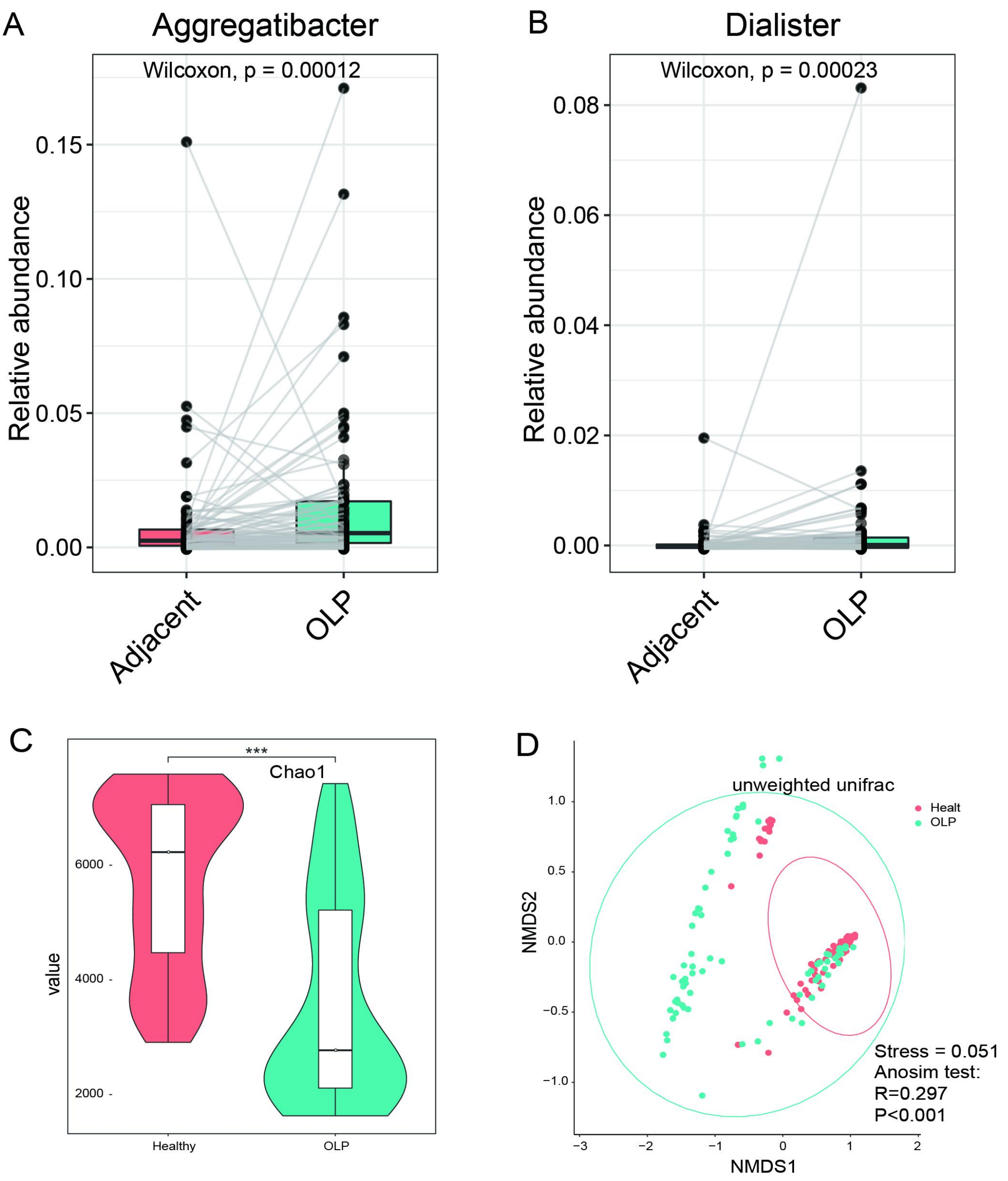

Figure S3 Screening of different species among the three groups. (A) The difference of Aggregatibacter between OLP and adjacent group. (B) The difference of Dialister between OLP and adjacent group. (C) The violinplot of Chao1 index between OLP and healthy group. (D) NMDS analysis based on unweighted unifrac distance between OLP and healthy group.

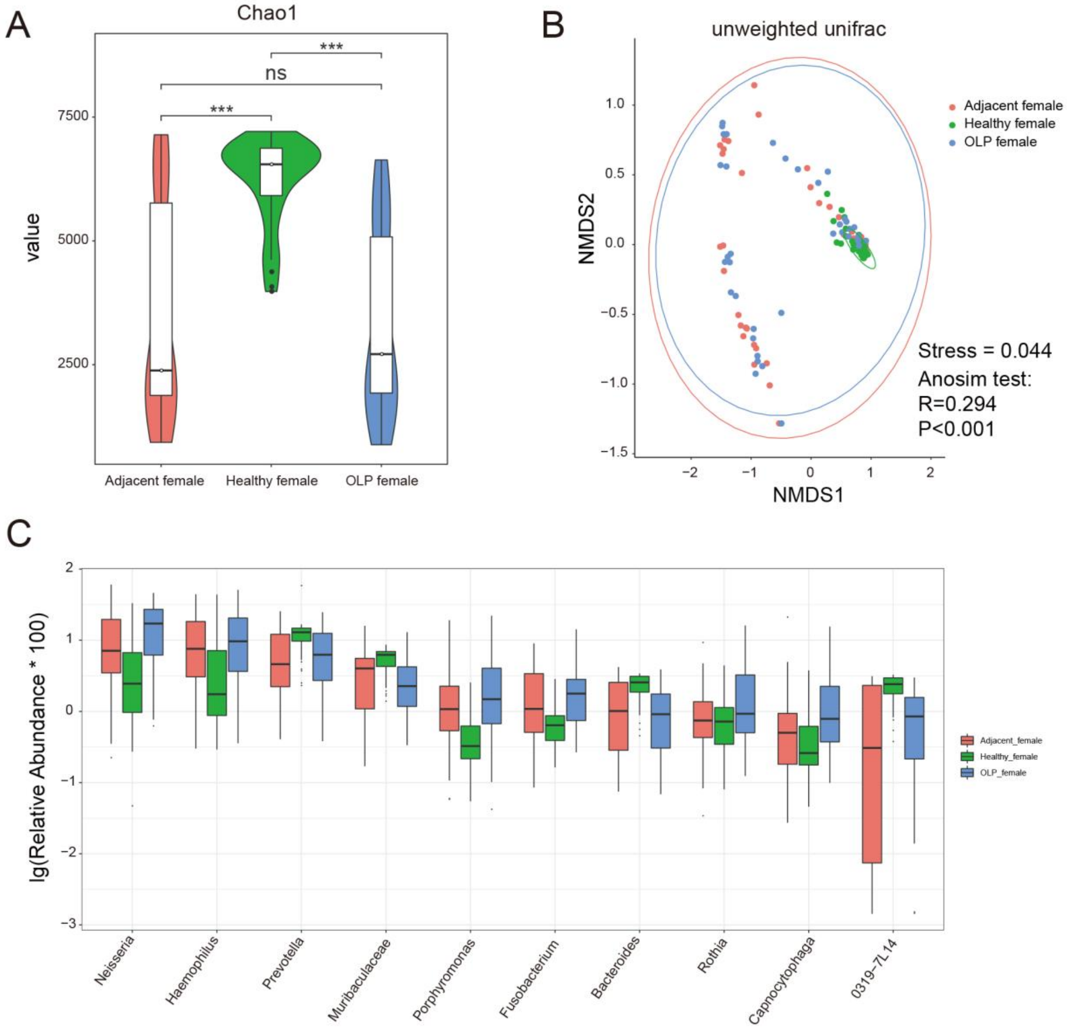

Figure S4 Comparison between female in three groups. (A) The violinplot of Chao1 index. (B) NMDS analysis based on unweighted unifrac distance. (C) Top10 different species at genus level among the three groups.

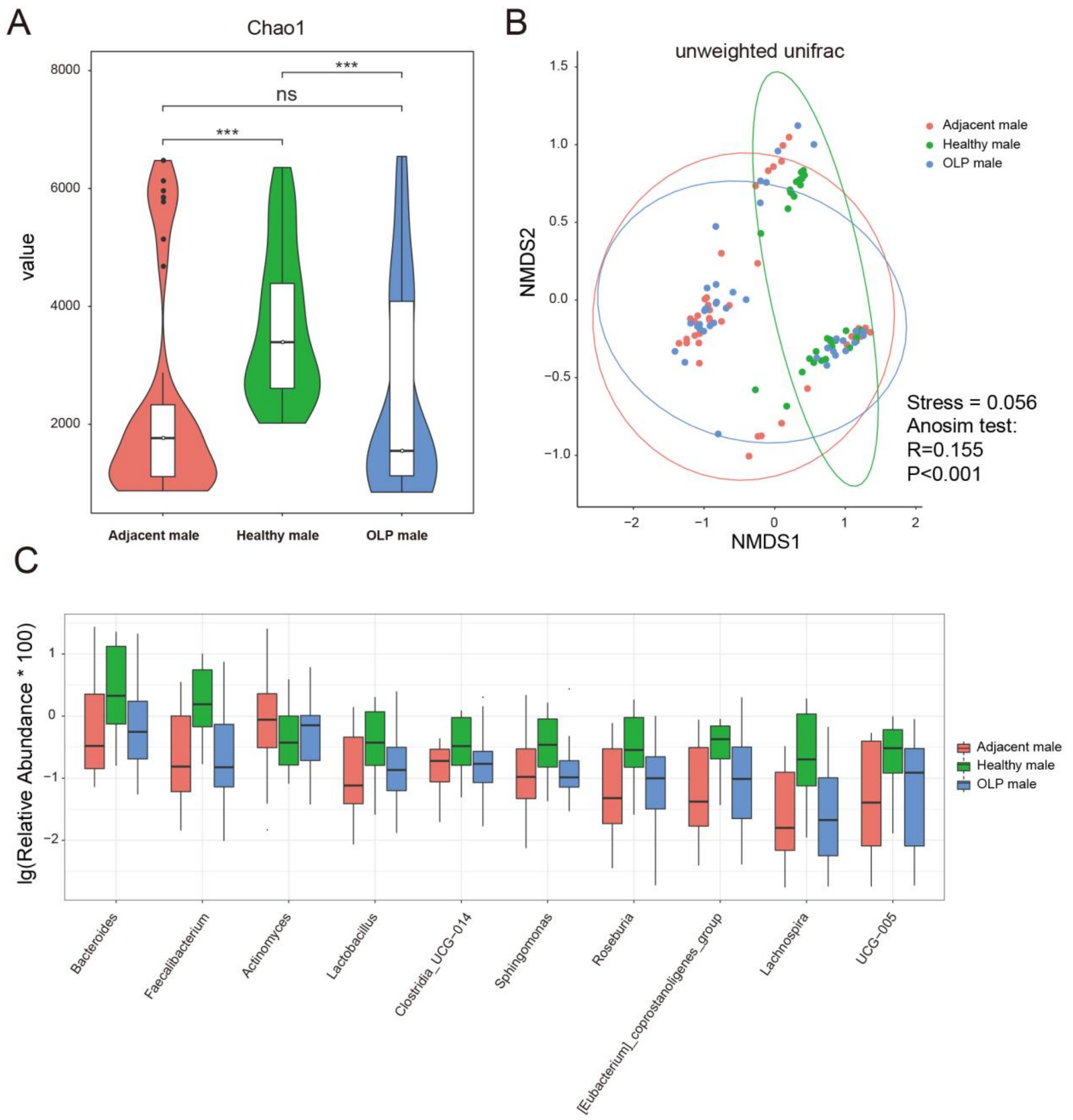

Figure S5 Comparison between male in three groups. (A) The violinplot of Chao1 index. (B) NMDS analysis based on unweighted unifrac distance. (C) Top10 different species at genus level among the three groups.

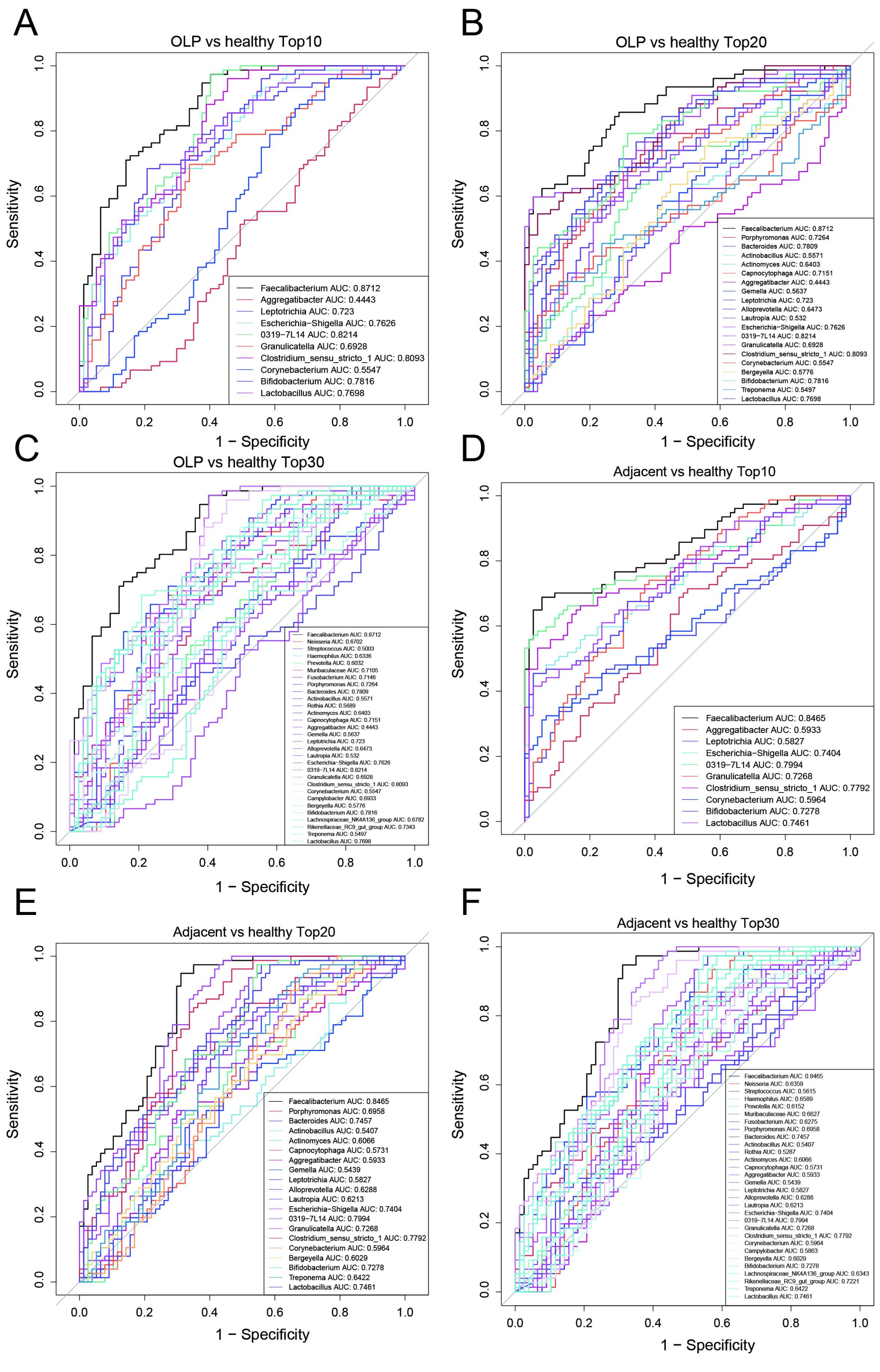

Figure S6 The top10, top20, and top30 taxa of the ROC curve. (A) The TOP 10 taxa of the ROC curve (OLP Vs. healthy group). (B) The TOP 20 taxa of the ROC curve

(OLP Vs. healthy group). (C) The TOP 30 taxa of the ROC curve (OLP Vs. healthy group). (D) The TOP 10 taxa of the ROC curve (adjacent vs. healthy group). (E) The TOP 20 taxa of the ROC curve (adjacent vs. healthy group). (F) The TOP 30 taxa of the ROC curve (adjacent vs. healthy group).
